# Maize Milling Method Affects Growth and Zinc Status but Not Provitamin A Carotenoid Bioefficacy in Male Mongolian Gerbils^1^,^2^,^3^,^4^

**DOI:** 10.3945/jn.116.241935

**Published:** 2017-02-01

**Authors:** Bryan M Gannon, Kevin V Pixley, Sherry A Tanumihardjo

**Affiliations:** 5 Interdepartmental Graduate Program in Nutritional Sciences, Department of Nutritional Sciences, and; 6 Department of Agronomy, University of Wisconsin–Madison, Madison, WI; and; 7 International Maize and Wheat Improvement Center, Texcoco, Mexico

**Keywords:** bioconversion, iron, retinol, vitamin A, pancreas

## Abstract

**Background:** Vitamin A (VA) and zinc deficiencies are prevalent. Maize is a common staple, and milling affects nutrient and nutrient-modifier profiles.

**Objective:** We investigated the interaction of maize milling methods (i.e., whole grain compared with refined) in male Mongolian gerbils aged 29–35 d with conventionally bred provitamin A–biofortified (orange) or white maize on VA and zinc status.

**Methods:** Study 1 (*n* = 67) was a 2 × 3 milling (whole compared with refined) by VA [no–vitamin A placebo group (VA−), orange, and VA-supplemented group (VA+)] design, with 4 wk of VA depletion followed by six 4-wk treatments (*n* = 10/treatment). Study 2 (*n* = 33) was a 2 × 2 milling-by-zinc [no-zinc placebo group (Zn−) compared with zinc-supplemented group (Zn+)] design, including 2 wk of VA depletion followed by four 3-wk treatments (*n* = 8–9/treatment). For study 1, positive and negative control groups were given supplemental VA at equimolar amounts to β-carotene equivalents consumed by the orange groups (74 ± 5 nmol/d) or placebo, respectively. For study 2, positive and negative control groups were given 152 μg Zn/d or placebo, respectively.

**Results:** Milling significantly affected zinc concentration, providing 44–45% (whole grain) or 9–14% (refined) NRC requirements. In study 1, orange maize improved liver VA concentrations (mean ± SD: 0.28 ± 0.08 μmol/g) compared with the white maize groups (0.072 ± 0.054 μmol/g). Provitamin A bioefficacy was similar. In study 2, neither zinc nor milling influenced liver retinol. Refined Zn− gerbils weighed less than others by day 14 (46.6 ± 7.1 compared with 56.5 ± 3.5 g, respectively; *P* < 0.0001). Milling affected pancreas zinc concentrations (refined Zn−: 21.1 ± 1.8 μg Zn/g; whole Zn−: 32.5 ± 5.8 μg Zn/g).

**Conclusions:** Whole-grain intake improved zinc and did not affect provitamin A bioefficacy. Other factors affected by milling (e.g., shelf life, preference, aflatoxin fractioning) need to be considered to maximize health.

## Introduction

Micronutrient deficiencies, including vitamin A (VA)^8^ and zinc, are prevalent and lead to considerable morbidity and mortality worldwide. Approximately 30% of the world’s population has inadequate zinc intakes, contributing to 28 million disability-adjusted life years because of diseases attributable to zinc deficiency (1). The primary clinical effects of inadequate zinc intake are reduced growth, impaired immune function, and adverse pregnancy outcomes (2). Furthermore, 190 million preschool-aged children and 19.1 million pregnant women are estimated to be at risk for VA deficiency (VAD) (defined by serum retinol concentrations <0.7 μmol/L), further increasing morbidity, the risk of death, and the same clinical adverse effects as zinc inadequacy (3). Considering that the regional prevalence of zinc inadequacy and VAD have substantial overlap (1, 3), it is pertinent to address multiple micronutrients to maximize benefit in target populations.

Biofortification is a plant-breeding or agronomic strategy to increase the nutrient content of staple crops. Maize (*Zea mays*) is a target for increasing provitamin A carotenoids because populations at risk for VAD consume white maize varieties that are devoid of carotenoids as a staple food. Biofortified orange varieties have been shown to be efficacious in improving or maintaining VA status in gerbils and humans (4, 5). Zinc is also a target for biofortification (6); however, many factors affect the zinc content of crops and its bioavailability, including soil zinc content, plant genetics, and zinc absorption inhibitors (e.g., phytates, fiber) (7, 8).

In addition, the milling method affects numerous nutritional aspects of maize (among other crops), including zinc, fiber, fat, and amino acid contents (9). Zinc and fiber have several interactions with VA and provitamin A carotenoids in humans. For example, zinc is essential for retinol-binding protein synthesis, and fiber inhibits β-carotene absorption (10, 11). However, the interactions of VA and zinc status are not entirely clear, and it has been suggested that zinc is required for β-carotene bioconversion (12).

The liver is the primary storage site of VA; therefore, liver concentrations are the gold standard for VA status assessment because liver stores can be mobilized during times of low dietary intake (2). Serum retinol concentrations are homeostatically controlled over a wide range of liver reserves and thus are not a sensitive indicator of VA status (13). Although most zinc is found in bone and skeletal muscles (14), the liver and pancreas are part of the rapidly exchanging zinc pool in the body and respond to dietary zinc intake (14–16). The pancreas is implicated in regulating zinc homeostasis through zinc binding and transport proteins that control excretion into the gastrointestinal tract (17–20). Furthermore, pancreatic zinc concentrations have been shown to be more sensitive to dietary zinc intakes than liver in mice (19). In addition, King and Keen (14) have shown that fecal zinc concentrations indicate the homeostatic regulation of absorption and excretion and respond to differences in dietary intake.

Investigating the nutritional impact of the milling method is pertinent because it is a modifiable factor that could be readily implemented. Although milling affects various nutrients and nutrient modifiers within consumed foods, its effects on provitamin A carotenoid bioefficacy and zinc status must be established to inform biofortification and nutrition education efforts. This study investigates the interaction between the milling method of maize and provitamin A carotenoid content on liver VA and zinc status in Mongolian gerbils (*Meriones unguiculatus*), which are useful as models because they absorb and metabolize provitamin A carotenoids similar to humans (i.e., they partially cleave provitamin A carotenoids to meet VA needs and absorb extra provitamin A carotenoids intact) (21–23). Zinc tissue concentrations have also been studied in gerbils fed standard rodent diets (22, 24).

## Methods

### 

#### Maize.

The biofortified orange maize was developed at the International Maize and Wheat Improvement Center as part of the HarvestPlus biofortified maize research project (25). The orange maize was formed by making all possible crosses and mixing the resultant seed with the use of 5 maize lines bred for a high concentration (17–24 μg/g) of provitamin A, most of which is β-carotene. The seed was shipped from Mexico to Zambia. The grain of this orange maize variety was produced on a commercial farm in Central Province, Zambia. After being harvested, the grain was analyzed and had ∼18 μg β-carotene equivalents (β-CEs)/g. White maize was a common variety purchased from local farmers in Eastern Province, Zambia. Maize was processed according to refined or whole-grain milling methods by a small mill that serves a rural community. Refining maize removes the germ and pericarp. After being harvested, orange maize was stored in a freezer at −20 to −10°C for ∼5 mo until 2 wk before it was ground; white maize was harvested the same year, kept dry, and stored at room temperature for ∼4–5 mo until grinding. The ground maize meals were stored in a freezer at −20°C until being delivered to the University of Wisconsin–Madison (UW) for use in these studies.

#### Feed.

The feed was the same between studies and was formulated to be 50% maize by weight, with maize providing the only source of zinc and VA in the form of provitamin A carotenoids (**Supplemental Table 1**). Maize was referred to by its milling method and provitamin A content (white or orange): whole white (WW), refined white (RW), whole orange (WO), and refined orange (RO). All other constituents were constant. Macronutrient content consisted of ∼21.6% protein, 46.1% carbohydrates, and 6.8% fat by weight and provided 3.3 kcal/g, which met or exceeded the macronutrient proportions recommended by the NRC (26). Note, however, that refining maize may affect several nutritive and nonnutritive components, and the macronutrient profile could deviate from that which was estimated. The RO meal (16.4 ± 0.1 μg β-CEs/g) was mixed with the RW meal in a 78.4:21.6 (wt:wt) ratio to equalize it with the WO (12.9 ± 0.3 μg β-CEs/g) group for β-carotene concentration.

#### Gerbils.

Male Mongolian gerbils aged 29–35 d (study 1) or 31–34 d (study 2) (Charles River Laboratories) were housed in groups (2−3/cage) during VA depletion and treatment (2–3/cage) in hanging wire-bottom cages to prevent coprophagy and mineral recycling. Animal handling procedures were approved by the UW College of Agricultural and Life Sciences Animal Care and Use Committee. Gerbils were weighed daily for 2.5 wk and 3 times/wk thereafter. Feed intake was measured daily by weighing the provided feed and subtracting the remaining and wasted feed. Room temperature and humidity were held constant with a 12-h light/dark cycle.

#### Study designs.

For study 1, gerbils were fed RW (*n* = 33) or WW (*n* = 34) during VA depletion (days 0–27) and received 50 μL cottonseed oil 3 times/wk to acclimate them to dosing procedures. After 28 d, 7 gerbils were killed while under isoflurane. The remaining gerbils were weight-matched and allocated into groups (*n* = 10/group) for the treatment period (days 28–56) such that gerbils remained on feeds with the same milling method. White maize (RW and WW) included positive and negative VA control groups: the VA-supplemented group (VA+) was given VA equimolar to β-carotene consumed in orange groups the previous day as retinyl acetate in ∼50 μL cottonseed oil, and the no-VA placebo group (VA−) and orange groups were given the same volume of placebo oil. Daily doses were administered orally with a positive displacement pipette. All gerbils were killed after 28 d of treatment (day 56).

For study 2, gerbils were fed RW (*n* = 17) or WW (*n* = 16) during depletion (study days 0–15). Zinc-supplemented (Zn+) groups (refined Zn+ or whole Zn+) received 152 μg Zn/d as aqueous ZnSO_4_, and no-zinc placebo (Zn−) groups (refined Zn− or whole Zn−) received a placebo (both 50 μL orally) with added sucrose (6% wt:vol) to increase palatability. After depletion, all gerbils were switched to orange maize with the same milling method as during depletion and continued receiving either zinc or the placebo for 21 d. All gerbils were killed on day 36. The liver, pancreas, and serum were stored at −80°C until analysis. Femur length was measured with the use of digital calipers. Both studies are shown in **Supplemental Figure 1**.

#### Carotenoid, retinol, and zinc analyses.

All samples were analyzed on a wet-weight basis, and data are reported as such. All sample analyses for carotenoids and retinol were performed under gold fluorescent lights to prevent photo-oxidation and isomerization. Feeds (*n* = 3/feed) were analyzed for carotenoids with the use of a previously published procedure for extraction (27) with a modified HPLC system (28). Serum retinol was extracted with the use of a modified procedure (29). Briefly, 250 μL serum and 30 μL C23 β-apocarotenol were extracted twice with 300 μL hexanes, dried under nitrogen, and resuspended in 50 μL [75:25 methanol:dichloroethane (vol:vol)], 35 μL of which was injected onto the HPLC system. Liver total retinol was analyzed by extracting and saponifying an aliquot with the use of a modified procedure (30). Modifications included the use of ∼0.25 g liver and 25 mL dichloromethane and injecting 20 μL (study 1) or 30 μL (study 2) of the reconstituted sample onto the HPLC system. The HPLC system for retinol analysis used a Gracesmart C18 (particle size, 5 μm; inside diameter, 4.6 mm; length, 250 mm) with 92:8 acetonitrile:water (vol:vol) at 1 mL/min (4) for study 1 and 95:5 acetonitrile:water (vol:vol) at 2 mL/min (30) for study 2. Liver β-carotene was analyzed with the use of an unsaponified fraction of the extract by HPLC (5, 27).

Zinc concentrations of the feed, liver, pancreas, and feces were determined with the use of inductively coupled plasma optical emission spectrometry by the UW Soil Testing Laboratory (study 1) or SoilNet LLC (study 2) by the same blinded technician following established protocols for animal (31) or plant (32) tissues. Feces were collected before baseline, midline, or final time points.

#### Fiber and phytate analysis.

Acid- and neutral-detergent fibers, dry matter, and ash for each feed type (*n* = 3 or 4/feed) were analyzed as previously described (33, 34). Phytates were analyzed for each feed type (*n* = 3) with the use of a modified colorimetric method (35) and quantified against standard 98% sodium phytate (Santa Cruz Biotechnology) (36).

#### Gerbil fur color.

Color space coordinates on the CIELab scale of the back fur of gerbils in study 2 were measured with the use of a portable Konica Minolta Chroma Meter CR-300 colorimeter. The CIELab L* scale ranges from 0 (black) to 100 (white), the a* scale ranges from −60 (green) to 60 (red), and the b* scale ranges from −60 (blue) to 60 (yellow). The total color difference was calculated as the change from baseline as follows:


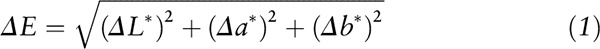


#### Statistical analysis and calculation of bioconversion factors.

Values are reported as means ± SDs. Data were analyzed with the use of SAS version 9.4 (SAS Institute). Outcomes of interest were evaluated with the use of independent 2-sample, 2-tailed *t* tests, linear regression, or 1-, 2-, or 3-factor ANOVA (including all interactions) as appropriate with the use of a general linear model. Effects of time were included for gerbil weights, feed intake, or fecal zinc concentrations taken over time (3-factor ANOVA); otherwise, data were analyzed at baseline, midline, or final time points. Post hoc letter groupings among treatment groups were determined with the use of least significant differences with the PDMIXED macro (37). Normality of residuals was tested with the use of the Shapiro-Wilk test; homogeneity of variance was tested with the use of Levene’s test. Data that failed normality or variance assumptions were analyzed nonparametrically with the use of rank-transformed data in the general linear model. Phytate and zinc feed data were randomized to calculate phytate:zinc molar ratios (*n* = 3/feed), variances, and *P* values. *P* < 0.05 was considered significant. Bioconversion factors in study 1 for orange maize groups were calculated in reference to the VA− and VA+ groups within milling groups (28).

## Results

### 

#### Feed properties.

By design, carotenoid, mineral, fiber, and phytate concentrations in the feeds had the expected relations (**Table 1**). Provitamin A carotenoid concentrations did not differ between the orange maize feeds but were much higher than feeds containing white maize. Orange maize provitamin A was predominantly β-carotene (∼97.6%) with some β-cryptoxanthin (∼1.7%) and α-carotene (∼0.7%); proportions were similar between WO and RO feeds. Zinc concentration was affected by milling; no significant differences existed within milling groups, but whole-milled feeds had 3–5-fold higher zinc concentrations than refined feeds.

**TABLE 1:** Nutrient concentrations and potential modifiers of interest in feed with 50% maize by weight fed to Mongolian gerbils^1^

	WW	WO	RW	RO
β-Carotene equivalents,^2^ μg/g	ND^c,^^3^	5.95 ± 0.65^a^	0.059 ± 0.002^b^	5.94 ± 0.19^a^
Zinc, μg/g	10.9 ± 0.90^a^	11.2 ± 0.33^a^	2.28 ± 0.19^b^	3.41 ± 0.76^b^
Iron,^4^ μg/g	62.8 ± 2.35^a^	49.9 ± 1.98^b^	40.9 ± 1.05^c^	40.5 ± 3.54^c^
Copper, μg/g	7.3 ± 1.2	8.6 ± 1.8	6.4 ± 0.9	6.8 ± 2.0
Neutral detergent fiber,^5^ % organic matter	7.61 ± 0.41^b^	9.01 ± 0.12^a^	4.64 ± 0.56^d^	6.53 ± 0.22^c^
Acid detergent fiber,^5^ % organic matter	4.92 ± 0.19^a^	5.18 ± 0.22^a^	3.80 ± 0.24^c^	4.35 ± 0.26^b^
Dry matter, %	90.5 ± 0.224^a^	88.1 ± 0.061^c^	90.1 ± 0.045^b^	88.0 ± 0.213^c^
Ash, %	4.33 ± 0.030^b^	4.45 ± 0.009^a^	3.91 ± 0.010^c^	3.89 ± 0.03^c^
Phytate, mg/g	5.58 ± 0.01^b^	6.49 ± 0.36^a^	1.89 ± 0.16^c^	2.26 ± 0.13^c^
Phytate:zinc molar ratio^6^	57.4 ± 3.0^b^	51.2 ± 4.4^b^	82.6 ± 10.3^a^	67.8 ± 14.7^a,b^

1 Values are means ± SDs. *P* values were determined by testing the null hypothesis that each variable is equal among treatment groups with the use of ANOVA. All *P* values were <0.0001 except those for the phytate:zinc molar ratio (*P* = 0.017) and copper (*P* = 0.38). Values in a row without a common superscript letter differ: a < b < c. ND, not detected; RO, refined orange; RW, refined white; WO, whole orange; WW, whole white.

2 β-carotene equivalent mass was determined by summing β-carotene isomers + 0.5 × α-carotene + 0.5 × β-cryptoxanthin × 536.9 (molar mass β-carotene)/552.8 (molar mass β-cryptoxanthin). Letter comparisons were made with the use of nonparametric analysis because of nonnormality with ND values; when excluded, statistical assumptions were satisfied for reporting.

3 The limit of detection was 0.005 μg/g.

4 Iron was provided in the mineral supplement as ferrous sulfate heptahydrate to provide 40 μg Fe/g.

5 Values for fiber are presented as % organic matter (subtracting ash weight) in g fiber/g organic matter × 100.

6 Phytate and zinc data were randomized to estimate variances and *P* values.

#### Feed intake and gerbil body and liver weights.

For study 1, gerbils given refined maize consumed less during each week of the depletion phase (days 0–27; *P* < 0.0001) (**Supplemental Figure 2**). Overall, intakes decreased during the treatment phase (days 28–56; *P* = 0.0038); the effects of milling, VA source, or their interactions were not significant (*P* ≥ 0.08).

For study 2, weekly feed intake was significantly affected by time, milling, zinc, and milling-by-time and milling-by-time-by-zinc interactions. Interactions were reflected in differences in treatment groups by week; groups did not significantly differ from one another at weeks 1 and 5 but did differ from weeks 2 to 4, with the refined Zn− group having intakes lower than all other groups during weeks 2 and 3 and the whole Zn+ group having higher intakes than all other groups during week 4.

**FIGURE 1:**
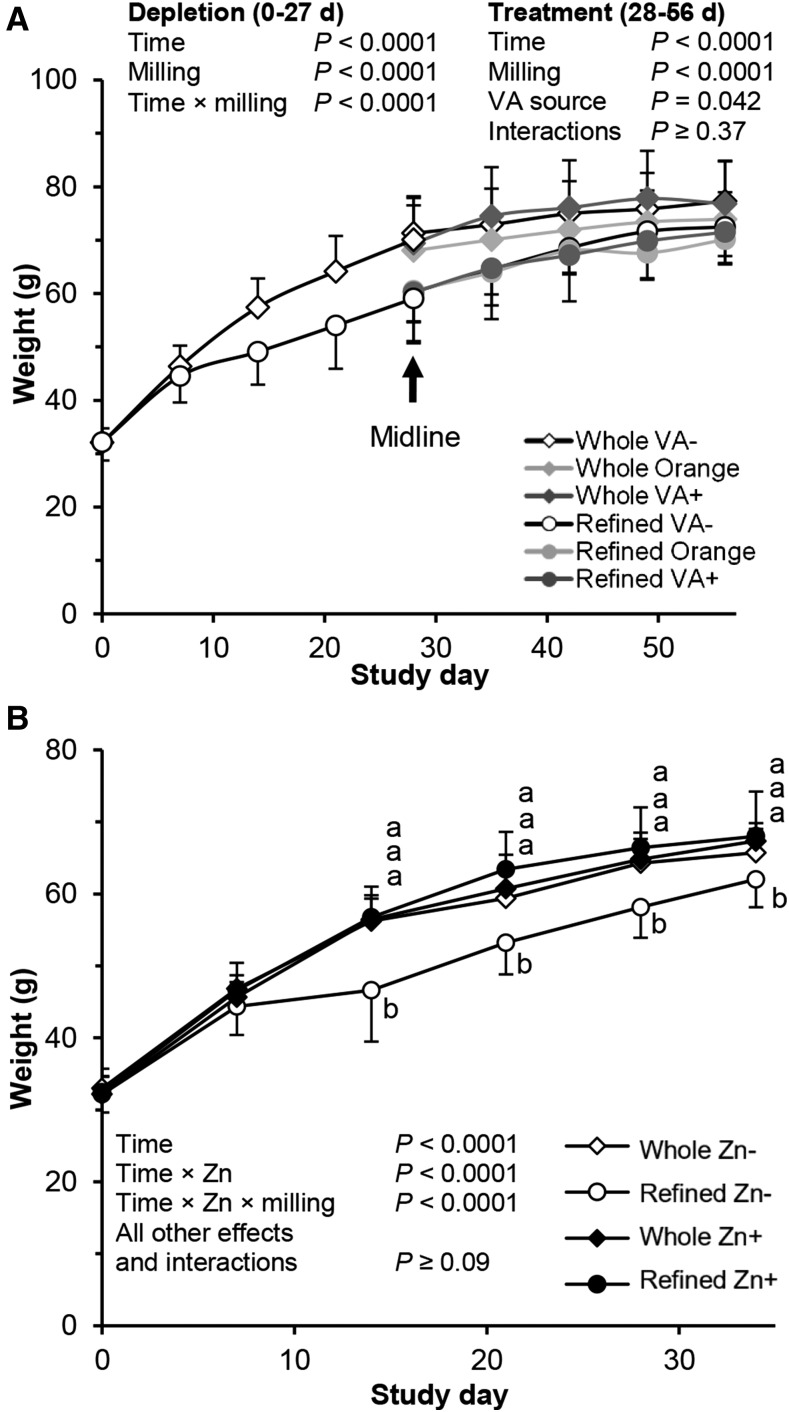
Gerbil body weight plotted against study day for gerbils that were fed 50% maize by weight feed for study 1 with VA+ and VA− groups (A) and study 2 with Zn+ and Zn− groups (B). All values are means ± SDs. (A) Gerbils in study 1 were aged 29–35 d on day 0. Sample sizes during depletion (days 0–27) were whole-milled white maize (*n* = 34) and refined-milled white maize (*n* = 33). Seven gerbils were killed on study day 28, after which the remaining gerbils were further randomized and weight-matched into treatments (*n* = 10/group). The VA+ group was given an oral dose of retinyl acetate daily in cottonseed oil. Orange and VA− groups received vehicle oil only. Data were analyzed as 2- (depletion) or 3-factor (treatment) ANOVA, including all interactions. (B) Gerbils in study group 2 were aged 31–34 d on day 0. Sample sizes were refined Zn− (*n* = 9). The Zn+ groups (*n* = 8/group) were given an oral dose of 152 μg Zn as aqueous ZnSO_4_ daily. The Zn− groups received vehicle only. Data were analyzed as 3-factor ANOVA, including all interactions. *P* < 0.05 was considered significant. VA, vitamin A.

For study 1, gerbil body weights (BWs) upon arrival did not differ among groups, but there were significant effects of milling on BW during the depletion and treatment periods (*P* ≤ 0.0001) (**Figure 1**). Changes in BW from midline (day 28) to endline (day 56) revealed an effect of milling (*P* = 0.026) but no effect of VA or an interaction, reflecting some catch-up growth by the refined groups. However, final BWs showed a milling effect in which refined groups were lighter (*P* = 0.0053), but neither an effect of VA source nor interaction existed. Study 2 groups had similar BWs upon arrival, but time and time-by-zinc and time-by-zinc-by-milling interactions affected BW; all other effects and interactions were not significant. Refined Zn− group gerbils weighed significantly less than all other groups from study day 14 through the remainder of the study (*P* ≤ 0.020); however, other groups did not differ at any time point.

In study 1, liver weights were affected by the VA source (VA−: 2.76 ± 0.25 g; orange and VA+: 2.48 ± 0.39 g; *P* = 0.020) but not by milling or an interaction. Relative liver weight (percentage of BW) was also affected by the VA source (VA−: 3.7% ± 0.3%; orange and VA+: 3.4% ± 0.4%; *P* = 0.016) but not by milling or an interaction. In study 2, neither liver weights (2.4 ± 0.3 g) nor relative liver weights (3.7% ± 0.3%) had effects of zinc, milling, or a zinc-by-milling interaction. Femur lengths (22.7 ± 0.6 mm) were not affected by zinc (*P* = 0.57), milling (*P* = 0.10), or a zinc-by-milling interaction (*P* = 0.10).

#### Liver retinol and β-carotene concentrations.

**FIGURE 2:**
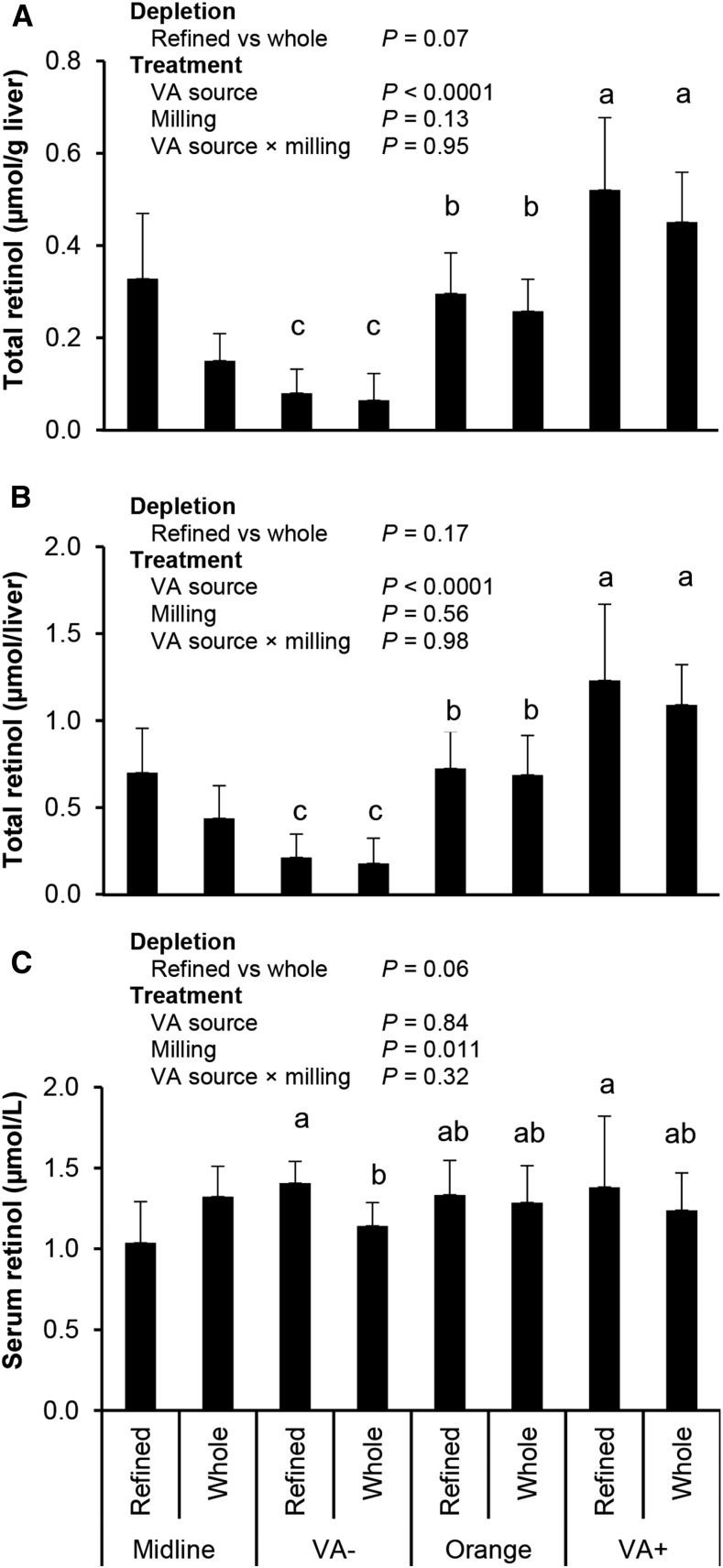
Liver retinol concentration (A), content (B), and serum retinol concentration (C) in male gerbils fed maize with different VA and milling treatments (study 1). Liver samples were saponified during the analysis; therefore, retinol represents (retinol + retinyl esters) present in the liver. All groups included 10 gerbils for final measurements except the midline whole (*n* = 4) and refined (*n* = 3) groups. All values are means ± SDs. Treatment data were analyzed with the use of 2-factor ANOVA. Groups without common letters were significantly different: a > b > c. *P* < 0.05 was considered significant. Midline statistical difference between refined and whole groups was determined with the use of a 2-sided *t* test. VA, vitamin A.

In study 1, liver retinol concentration (retinol + saponified retinyl esters) and total retinol did not differ at midline (**Figure 2A, B**). The VA source influenced liver VA values, whereas the milling method and interaction were not significant. In study 2, neither liver retinol concentration (0.43 ± 0.12 μmol liver/g) nor total retinol (1.03 ± 0.27 μmol/liver) were affected by zinc, milling, or a zinc-by-milling interaction (*P* ≥ 0.10).

In study 1, liver β-carotene concentration and total liver β-carotene were greater in the RO than WO groups (**Table 2**). For study 2, liver β-carotene concentration and total liver β-carotene were affected by milling (refined > whole) but not zinc or an interaction.

**TABLE 2:** Liver β-carotene content of Mongolian gerbils given feed with 50% maize by weight with different milling methods^1^

	Refined orange Zn−	Whole orange Zn−	Refined orange Zn+	Whole orange Zn+	*P*
Study 1					
Liver concentration, nmol β-carotene/g	1.21 ± 0.37	0.47 ± 0.20	—	—	<0.00010
Total liver β-carotene, nmol	3.00 ± 1.08	1.23 ± 0.46	—	—	0.00015
Study 2					
Liver concentration, nmol β-carotene/g	2.40 ± 0.969^a^	1.10 ± 0.515^b^	1.71 ± 0.856^a,b^	1.56 ± 0.872^a,b^	
Milling					0.018
Zinc					0.69
Milling by zinc					0.057
Total liver β-carotene, nmol	5.53 ± 2.00^a^	2.81 ± 1.50^b^	4.31 ± 2.41^a,b^	3.63 ± 1.88^a,b^	
Milling					0.026
Zinc					0.88
Milling by zinc					0.19

1 Data are means ± SDs, *n* = 10/group (study 1) and 8–9/group (study 2). Values in a row without a common superscript letter do not differ: a < b. *P* values were determined with the use of independent 2-sample, 2-tailed *t* tests (study 1) or 2-factor ANOVA (study 2).

#### Bioconversion factors.

From study 1, the bioconversion factors for the orange groups were calculated to be 3.6 and 3.7 μg β-CE:1 μg retinol for WO and RO, respectively.

#### Serum retinol concentrations.

Serum retinol concentrations were not affected by the VA source or a VA source-by-milling interaction; however, milling significantly affected serum retinol concentrations, with refined group concentrations (1.37 ± 0.24 μmol/L) significantly higher than whole-milled group concentrations (1.22 ± 0.20 μmol/L) (Figure 2C). One gerbil had a serum retinol concentration <0.7 μmol/L (group RW; VA+), whereas all other gerbils had serum retinol concentrations >0.7 μmol/L, which is considered normal for mammals. In study 2, serum retinol concentrations were not affected by zinc (*P* = 0.081), milling, or a zinc-by-milling interaction.

#### Zinc analyses.

**FIGURE 3:**
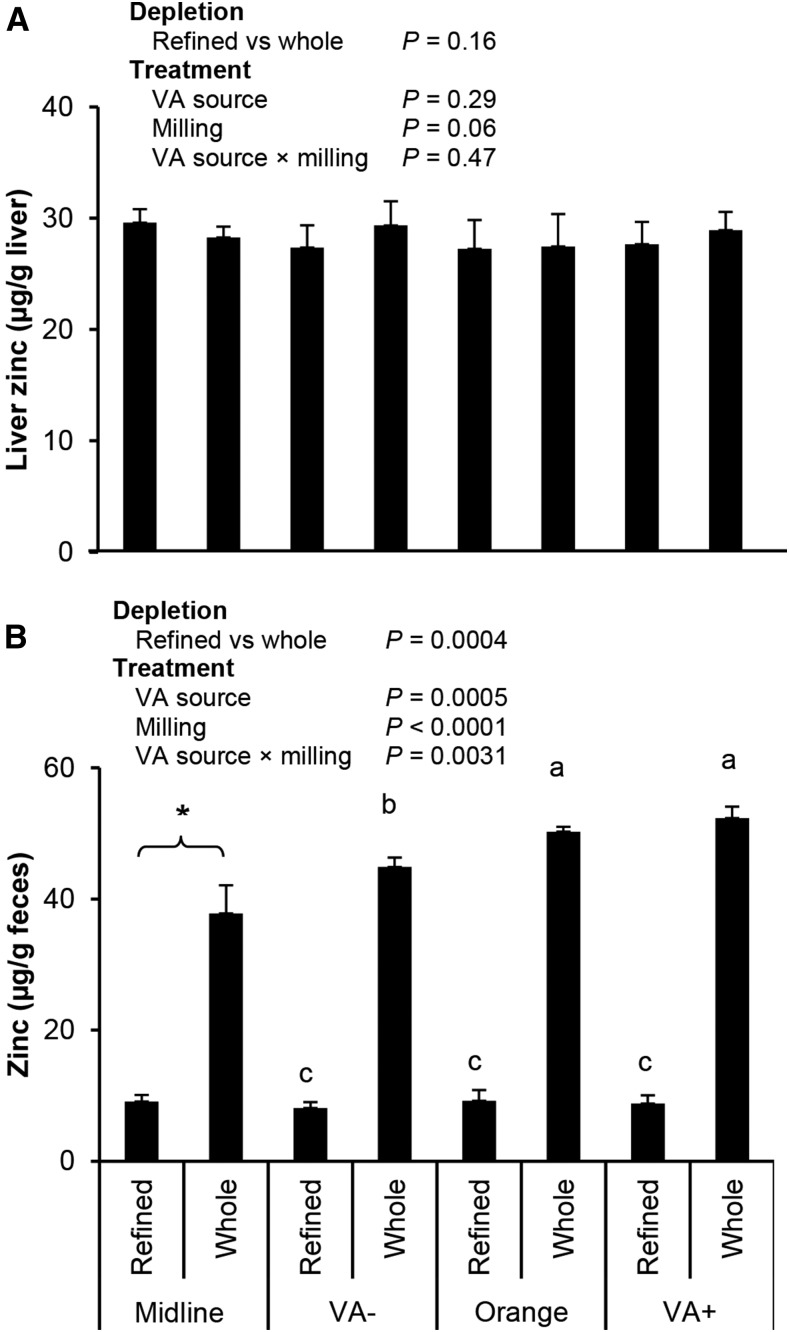
Liver (A) and fecal (B) zinc concentrations in male gerbils fed maize with different VA and milling treatments (study 1). All groups shown in panel A included 10 gerbils at final measurements except midline whole (*n* = 4) and refined (*n* = 3). Fecal zinc concentration was pooled from the feces of each treatment group (*n* = 3/group) at the preceding midline (day 28) or final (day 56) time points. Treatment data were analyzed with the use of 2-factor ANOVA. Treatment groups without common letters are significantly different: a > b > c. *P* < 0.05 was considered significant. *Midline statistical difference between refined and whole groups was determined with the use of a 2-sided *t* test. VA, vitamin A.

**FIGURE 4:**
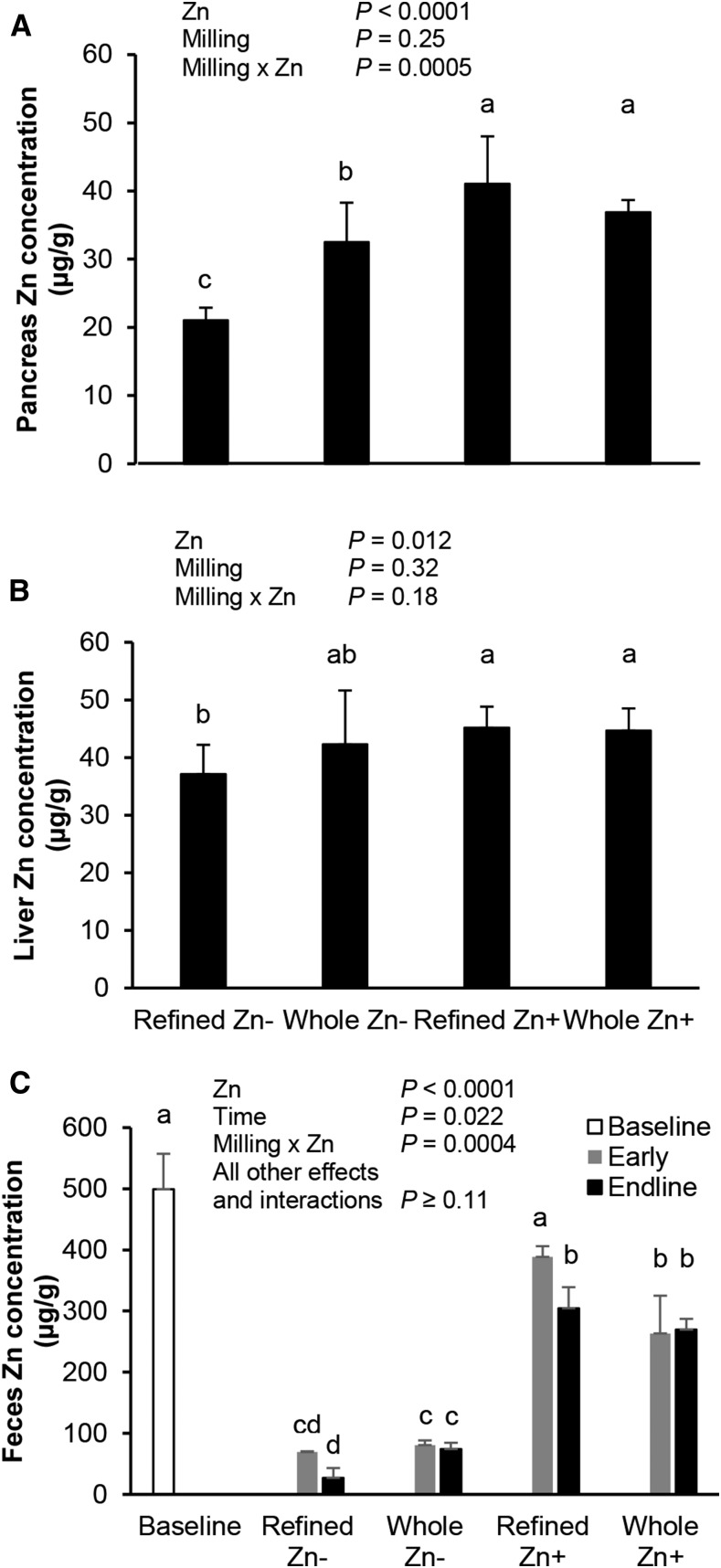
Pancreatic zinc (A), liver (B), and fecal (C) zinc concentrations in male gerbils fed maize with different milling and supplemental zinc treatments (study 2). All liver zinc groups included 8 gerbils except the refined Zn− group (*n* = 9) at final measurements, and all fecal zinc groups included 8 gerbils except the whole Zn− group (*n* = 7) at final measurements. Fecal zinc concentration was pooled from the feces of gerbils from each treatment group (*n* = 3/group) at baseline (day 0), early (day 2–3), or endline (day 35) time points. All values are means ± SDs. Data were analyzed with the use of 2-factor (for the pancreas and liver) and 3-factor (for feces to include time) ANOVAs, including all interactions. Treatment groups with uncommon letters are significantly different: a > b > c > d. *P* < 0.05 was considered significant.

In study 1, liver zinc and total liver zinc did not differ at midline (day 28) (**Figure 3**). After treatment, liver zinc concentrations had a nonsignificant trend toward a milling effect and were not affected by the VA source or a VA source-by-milling interaction. Total liver zinc was not affected by treatment, and there were no interactions (overall mean: 72.7 ± 15.5 μg). The milling method drastically affected fecal zinc concentration (whole > refined). In study 2, dietary zinc and a zinc-by-milling interaction, but not milling, affected pancreatic zinc concentrations (**Figure 4**). Dietary zinc but not milling or the zinc-by-milling interaction affected zinc concentrations in the liver. Liver and pancreatic zinc concentrations were positively and significantly correlated (*P* = 0.0022; *r*^2^ = 0.28). At baseline (day 0), the fecal zinc concentration was relatively high. It responded rapidly to zinc intake in all groups during days 2–3 and was either maintained or reduced by endline (day 35).

#### Iron and copper analyses.

Liver iron concentration from study 1 was not different between RW and WW groups after depletion (data not shown). After treatment, liver iron concentration was significantly affected by milling (RW: 122 ± 34 μg Fe/g liver; WW: 139 ± 27 μg Fe/g liver; *P* = 0.033) but not the VA source or an interaction. Liver copper concentrations were not affected by treatment during depletion or treatment (overall mean: 6.1 ± 0.9 μg/g).

#### Gerbil fur color.

In study 1, gray patches of fur were observed in all gerbil groups that were fed refined maize. The study 2 gerbil fur L* value (lightness) had a time-by-milling interaction; refined maize groups’ fur had increasing lightness with time compared with whole maize groups (**Supplemental Figure 3**); a* and b* values had time-by-milling-by-zinc interactions; and total color difference (Equation *1*) had a milling-by-zinc interaction (*P* ≤ 0.034) but did not differ at endline among treatment groups.

## Discussion

We conducted 2 studies to evaluate the milling method in Mongolian gerbils to provide evidence that biofortified orange maize bioefficacy is not altered by the milling method or supplemental zinc. Study 1 included VA+ and VA− groups to determine the bioconversion factor (μg β-CE:1 μg retinol) for β-carotene in the orange maize, which was similar between orange groups: WO (3.6) and RO (3.7). These bioconversion factors were in the range of other gerbil studies with maize containing adequate zinc (30 μg feed/g), i.e., 2.4–6.3 μg β-CE:1 μg retinol (5, 28, 38, 39). Study 2 included Zn+ and Zn− groups to evaluate differences caused by zinc intakes; liver VA concentrations were not affected by milling or zinc. These data suggest that even in the refined feeds, the low concentration of zinc did not interfere with β-carotene bioefficacy in gerbils. Furthermore, bioconversion was not influenced by other compounds that may be affected by the milling method, such as fiber and fat. Fat is a known promoter of β-carotene absorption and bioconversion, whereas fibers are inhibitory (40, 41). Nonetheless, milling and supplemental zinc did affect other zinc outcomes.

Impaired growth is the primary clinical sign of zinc deficiency, and milling effects or interactions on BW were seen during the depletion and treatment phases in both studies, indicating that the milling method, in the context of feeds deficient in Zn− and VA−, was sufficient to cause phenotypic differences in the gerbils (Figure 1). Little data to our knowledge exist on zinc requirements in gerbils, but 11 μg feed/g from the whole-grain meals matched growth in gerbils that were given the same feed with 152 μg supplemental Zn/d in study 2, which was based on NRC requirements of 25 μg feed/g and estimated daily feed intake. Furthermore, a phenotypic difference in fur coat color was observed in all study 1 treatment groups based on the milling method, whereby prolonged refined maize intake resulted in gray patches of fur compared with standard brown. This observation was assessed in study 2 with the use of a colorimeter to measure changes and differences in fur color (Supplemental Figure 3). Although some milling, zinc, and time interactions significantly affected fur color, including lightening of the groups that were fed refined maize with time, the same magnitude of difference among treatment groups as subjectively observed in study 1 did not occur, likely because of the shorter duration of study 2. Thus, we were not able to definitively determine whether this was caused by reduced zinc intake alone or from other changes during the refining process.

In both studies, gerbil BW differences during the depletion period were reflected by a difference in feed intake, which lasted through week 4 of both studies (Supplemental Figure 2). In study 2, the refined Zn− group had the lowest intakes, and a significant time-by-milling-by-zinc interaction indicated that it was the lack of zinc that caused a reduction in feed intake, a known phenomenon in numerous species, including rats, swine, guinea pigs, and humans (42, 43). Zinc given orally to zinc-deprived rats increased feed intake through appetite-stimulating peptides coupled with the vagus nerve (44). The literature is inconsistent in regard to whether reduced growth during zinc deficiency is purely the result of reduced feed intake or whether the lack of zinc inhibits the growth process. Pair-fed zinc-adequate gerbils grew faster than zinc-deficient gerbils, but other studies found no difference between groups (42). Regardless, low zinc feeds prepared from 50% refined maize decreased both feed intake and BW in these studies.

Liver and pancreatic zinc concentrations were used as markers of zinc status (19, 20). In study 2, both outcomes were affected by zinc, and the pancreas had a milling-by-zinc interaction. The refined Zn− group had the lowest zinc concentrations, whereas Zn+ groups were not different from one another. The whole-grain Zn− group had higher pancreatic zinc concentrations than the refined Zn− group at endline. Thus, gerbil pancreatic zinc concentrations reflected differences in dietary zinc intake. Although liver zinc concentrations did not differ in study 1, they did respond to dietary zinc in study 2, which is likely because of more total zinc and the higher bioavailability of the aqueous zinc supplement (20). Liver zinc concentrations were similar to published values after a correction factor of 3.3 was used to account for the water weight of rodent livers (45) (∼30 μg/g; range of different feeds: 30–110 μg Zn/g) (22). One study reported higher values (∼130 μg/g; dietary feeds: 95 μg Zn/g) (24), but the values were not explicitly stated as being on a wet or dry basis. If on a dry basis, the values are close to others based on wet weight (∼39 μg Zn/g). The pancreas responded more to changes in dietary zinc intake than the liver, as seen in mice (19). These data indicate that the maize milling method can substantially alter zinc content, further affecting markers of zinc status. Although feed was iron-fortified at recommended concentrations, liver iron concentrations were affected by the milling method, which could affect iron outcomes if limited in the diet.

The primary zinc excretion route is from the intestine and reflects intake (2, 14). Fecal zinc concentration was affected by milling in study 1 and a zinc effect and milling-by-zinc interaction in study 2. Relative patterns of treatment groups at endline matched those of the pancreas: refined Zn− < whole Zn− < refined Zn+ = whole Zn+. Differences observed between refined Zn− and whole Zn− groups, despite being lower than the intake requirements suggested by the NRC (25 μg Zn/g) for feeds with a considerable amount of phytates (26), suggest that gerbils regulate absorption and excretion even between these marginal and deficient intake concentrations. This relation suggests that although the supplemental zinc had the greatest effect on outcomes, the milling method still affected markers of zinc intake and status.

Total absorbed zinc is a product of feed zinc concentration, feed intake, and zinc bioavailability. A positive relation was noted between zinc and phytate concentrations in the refined and whole-milled feeds; therefore, it is important to keep this interaction in mind when evaluating zinc outcomes. The phytate:zinc molar ratios in the refined feeds were higher than in whole-milled feeds; however, phytate:zinc molar ratios ≥20 have demonstrated a constant inhibitory effect (46), and therefore the inhibitory effect of phytates in this study (phytate:zinc molar ratio range: 51–83) would be constant, with only the milling method affecting feed zinc concentration and study outcomes.

Liver VA concentrations and comparisons by VA concentrations in feeds were as expected: VA+ > orange > VA− (5, 38). This is in agreement with other gerbils that were fed high β-carotene maize with a positive control group that were fed preformed VA on an equimolar basis (5). Both treatment groups that did not receive any form of VA had mean liver retinol concentrations <0.1 μmol/g [deficiency cutoff (13)], whereas all groups that received VA or orange maize were well above this cutoff. β-Carotene is present primarily in maize endosperm (47), which is consistent with our initial findings of RO maize having higher β-carotene concentrations than WO maize. Despite equalizing orange feeds for β-carotene, increased hepatic β-carotene was observed in the refined groups in both studies, but liver retinol concentration did not differ between whole and refined groups, likely indicating that gerbils that were fed refined grains absorbed more total β-carotene, whereas gerbils that were fed whole grains absorbed and cleaved sufficient β-carotene to meet VA needs.

The effect of milling on serum retinol concentrations in study 1 (refined > whole-milled) for the treatment phase in the absence of a VA effect or VA-by-milling interaction is surprising because the reverse may have been expected because of the potential reduction of retinol-binding protein synthesis with inadequate zinc (10, 48). However, during the depletion phase, a nonsignificant trend existed in the opposite direction, reflecting the homeostatic and variable nature of serum retinol to treatments (13, 49). Serum retinol concentrations in study 2 were not affected by treatment and were comparable to study 1 and other gerbil studies that used similar dietary provitamin A treatments with sufficient zinc (1.24 ± 0.19 to 1.37 ± 0.35 μmol/L) (28, 38). Perhaps a more severe zinc deficiency is needed to affect serum retinol concentrations than achieved in these studies.

This study complements a recent study of humans in Zambia (6) that determined the total daily absorption of zinc from biofortified high-zinc maize (34 μg Zn/g) compared with standard maize (21 μg Zn/g) for a single day of test meals with the use of stable isotope methodology. A whole-grain milling method was used because much of the zinc was lost if the grain was refined in an open hammer mill (reduced to 5 and 2 μg Zn/g flour, respectively). The standard maize was similar in zinc concentration to our whole-milled maize, and the authors noted an 85–90% reduction in zinc after polishing. The high-zinc variety also had a higher phytate content but lower phytate:zinc molar ratio than the standard maize, although both ratios were >20, likely indicating a constant inhibitory effect (46). The authors of the human study (6) concluded that high-zinc maize in humans yielded 83% more total daily zinc absorption than the standard maize (1.1 ± 0.5 compared with 0.6 ± 0.2 mg/d). The estimated physiologic requirement for children of this age is 0.744 mg/d (2); thus, high-zinc and standard maize provided 148% and 81%, respectively, of the physiologic requirement. The human study (6) and the current study demonstrate the importance of the milling method on the zinc concentration of ground maize and highlight the fact that whole-milled maize can supply substantial amounts of zinc when consumed as a staple food despite high phytate:zinc ratios.

This study indicates that the maize milling method used is sufficient to cause numerous adverse outcomes related to zinc status, including reduced growth, reduced feed intake, lower pancreatic zinc concentrations, and disparate fecal zinc concentrations, without affecting β-carotene bioefficacy. Because biofortification and other nutritional efforts continue to address the major worldwide micronutrient deficiencies of zinc, VA, and iron, it is pertinent to further investigate food processing and preparation methods because they pertain to multiple nutrients and compounds that interact with and affect the bioavailability of nutrients of interest. Nonnutritive factors must also be considered because there is evidence for an interaction between mycotoxins present in maize grain and the amount remaining in different milling fractions (50, 51). In Zambia, refined milling is typically preferred for smoother consistency, purer white color, and increased shelf life in suboptimal storage conditions. Furthermore, the waste from refining maize is fed to animals and therefore serves secondary nutritive purposes. These nonnutritive factors and cultural practices must all be considered to maximize overall health outcomes.

## Footnotes

8 Abbreviations used: BW, body weight; RO, refined orange; RW, refined white; UW, University of Wisconsin–Madison; VA, vitamin A; VAD, vitamin A deficiency; VA−, no–vitamin A placebo group; VA+, vitamin A–supplemented group; WO, whole orange; WW, whole white; Zn−, no-zinc placebo group; Zn+, zinc-supplemented group; β-CE, β-carotene equivalent.
